# Cystinosis symposium: a rare disease model for comprehensive care

**DOI:** 10.3389/fped.2025.1630897

**Published:** 2025-07-24

**Authors:** Ashley M. Gefen, Frederick J. Kaskel, Joshua J. Zaritsky

**Affiliations:** ^1^ Division of Nephrology, Department of Pediatrics, Phoenix Children's Hospital, Phoenix, AZ, United States; ^2^ Division of Nephrology, Department of Pediatrics, Children's Hospital at Montefiore, New York, NY, United States; ^3^ Division of Nephrology, Department of Pediatrics, Albert Einstein College of Medicine, Bronx, NY, United States

**Keywords:** cystinosis, rare disease, comprehensive care, transition, cystinosis metabolic bone disease

## Abstract

The time to provide a forum for collaboration in cystinosis research underscored the recent Cystinosis Symposium: A Rare Disease Model for Comprehensive Care held at the New York Academy of Medicine, on 31 May 2024, an event sponsored by the Cystinosis Research Network and numerous corporate entities (Amgen and others). This conference was one of the first to address the glaring deficiencies in the care of children and adults with cystinosis by focusing on themes that are mutually applicable to multidisciplinary healthcare providers, educators, and families. The ultimate goal was to disseminate new information and awareness to all engaged in the cystinosis community and provide an algorithm to navigate more effectively the promising future toward a cure.

Advances in our understanding of the cellular/molecular abnormalities in nephropathic cystinosis over the past 20 years have heralded three major obvious advances. First, the implementation of an effective treatment to interrupt and slow down the progression of the multisystem phenotypic expressions in cystinosis beginning in early life and extending throughout the pediatric journey. Second, the extension of patients with cystinosis reaching well into adulthood with its attendant challenges. Finally, the exciting potential to apply the newest science for gene therapies to eradicate this lifelong condition.

Despite these promising advancements in the care of children and adults with cystinosis, there remains substantial gaps and challenges in our knowledge base and ability to offer the most inclusive and beneficial care to patients and families. Earlier genetic recognition of the condition is possible but not fully implemented across the country. Concurrently, often the lack of recognition of the disease and its attendant multiorgan presentation results in late diagnosis and initiation of corrective treatments. Added to this gap is our inability to construct a team of healthcare professionals across the life course extending from primary care to a host of varied subspecialists, including social work, nutritionists, educators, and quality-of-life navigators.

Thus, the time to provide a forum for collaboration underscored the recent Cystinosis Symposium: A Rare Disease Model for Comprehensive Care held at the New York Academy of Medicine, on 31 May 2024, an event sponsored by the Cystinosis Research Network (CRN) and numerous corporate entities (Amgen and others). This conference was one of the first to address these glaring deficiencies in the care of children and adults with cystinosis by focusing on themes that are mutually applicable to multidisciplinary healthcare providers, educators, and families. The ultimate goal was to disseminate new information and awareness to all engaged in the cystinosis community and provide an algorithm to navigate more effectively the promising future toward a cure.

An introduction to rare disease: cystinosis as a model. Dr. William Gahl, one of the eminent National Institutes of Health (NIH) researchers who first reported on the gene defects responsible for lysosomal storage abnormalities in cystinosis, provided an overview of the field. His vast cumulative data on the outcomes of the multiple organ system derangements in cystinosis served as a template to track the journey of understanding progress made in the identification and early treatments for this disorder. He emphasized the importance of newborn screening for early detection.

The optimization of care for children with cystinosis has not been achieved for the preemptive recognition of multiple neurocognitive and developmental abnormal milestones, which offer significant challenges for each child/adult to achieve their expectations and quality of life. Neuroscientists who have made contributions to this area are Dr. John Foxe and Dr. Sophie Molhom, who have reported on unique neurological observations in children and adults with the disorder. Their recommendations for targeted behavioral and educational measures elicited an active discussion among the participants and attendees.

A patient and caregiver panel addressed “Challenges Within Rare Disease” and offered an opportunity for families to describe their personal experiences as patients and parents to exemplify some of the major hurdles and achievements in the care of cystinosis patients across the life course.

An often underrecognized comorbidity of cystinosis in childhood is the orthopedic, neuromuscular, and other manifestations of a myopathy that is progressive and debilitating. A pediatric orthopedist, Dr. Melinda Sharkey, with extensive experience in this area, reviewed an algorithm for detection and corrective measures. Dr. Reza Sadjadi, an expert in the myopathy associated with cystinosis, presented new information on biomarkers and treatments.

A major challenge to caregivers, patients, and parents is the gap in our knowledge as to how best engage in “transition of care” initiatives. All too often we learn about patients who have fallen through the gap once they age out of pediatrics and need an adult practitioner or specialist. Dr. Cybele Ghossein, an internal medicine nephrologist, has developed a unique outpatient experience with both pediatric and internal medicine specialists participating in the total care of these patients. A distinguished panel of pediatric and internal medicine specialists discussed their experiences with transition and a useful algorithm was presented for implementation in any institution.

Families often face isolation and despair especially at the initial diagnosis of cystinosis in a loved one, and now there is hope with the success of the CRN in supporting engagement and advocacy. The topic of Engagement and support in rare disease: CRN as a model will be discussed by leaders in the CRN organization and National Organization for Rare Disorders (NORD).

The epidemic of mental health disorders is problematic in all populations, while those with special conditions are particularly prone to these challenges. Mental health and quality of life was addressed by an expert in the field of rare diseases and cystinosis, Dr. Maya Doyle. Her perspective as a social worker throughout the life course served as a template for initiatives to engage networks of care providers to provide earlier recognition of mental health issues.

Novel genetic treatments of rare diseases were discussed by Dr. Stephanie Cherqui, a leading investigator of stem cell gene therapy clinical trials in cystinosis. In addition, another eminent investigator, Dr. Paul Goodyer, reviewed the seminal contributions that have advanced the preventive treatments for cystinosis at the gene level with promising prospects for the future.

Overall, the Symposium offered the unique opportunity for investigators, families, and diverse care providers to participate in a mutually beneficial and rewarding experience, which generated much discussion and elicited hope for the best possible treatment of patients with cystinosis.

